# Ryanodine Receptor Staining Identifies Viable Cardiomyocytes in Human and Rabbit Cardiac Tissue Slices

**DOI:** 10.3390/ijms241713514

**Published:** 2023-08-31

**Authors:** Ann-Katrin M. Pfeuffer, Linda K. Küpfer, Thirupura S. Shankar, Stavros G. Drakos, Tilmann Volk, Thomas Seidel

**Affiliations:** 1 Institute of Cellular and Molecular Physiology, Friedrich-Alexander University (FAU) Erlangen-Nuremberg, 91054 Erlangen, Germany; ann-katrin.pfeuffer@fau.de (A.-K.M.P.); linda.kuepfer@fau.de (L.K.K.); tilmann.volk@fau.de (T.V.); 2 Nora Eccles Harrison Cardiovascular Research and Training Institute, University of Utah, Salt Lake City, UT 84112, USA; u6009805@utah.edu (T.S.S.); stavros.drakos@hsc.utah.edu (S.G.D.)

**Keywords:** ryanodine receptor, cardiac tissue slices, death staining, sarcoplasmic reticulum, viability assay

## Abstract

In terms of preserving multicellularity and myocardial function in vitro, the cultivation of beating myocardial slices is an emerging technique in basic and translational cardiac research. It can be used, for example, for drug screening or to study pathomechanisms. Here, we describe staining for viable cardiomyocytes based on the immunofluorescence of ryanodine receptors (RyRs) in human and rabbit myocardial slices. Biomimetic chambers were used for culture and measurements of contractile force. Fixable fluorophore-conjugated dextran, entering cells with a permeable membrane, was used for death staining. RyRs, nuclei and the extracellular matrix, including the t-system, were additionally stained and analyzed by confocal microscopy and image processing. We found the mutual exclusion of the RyR and dextran signals in cultivated slices. T-System density and nucleus size were reduced in RyR-negative/dextran-positive myocytes. The fraction of RyR-positive myocytes and pixels correlated with the contractile force. In RyR-positive/dextran-positive myocytes, we found irregular RyR clusters and SERCA distribution patterns, confirmed by an altered power spectrum. We conclude that RyR immunofluorescence indicates viable cardiomyocytes in vibratome-cut myocardial slices, facilitating the detection and differential structural analysis of living vs. dead or dying myocytes. We suggest the loss of sarcoplasmic reticulum integrity as an early event during cardiomyocyte death.

## 1. Introduction

In the past few years, myocardial tissue culture has gained importance as an in vitro model of the heart. The ex vivo cultivation of living human and animal tissue slices for several days to months is possible under continuous electrical stimulation and a physiological preload [1,2,3,4]. Myocardial slices of approximately 200–500 µm thickness mounted in dedicated biomimetic cultivation chambers contract stably with a sustained multicellular environment consisting not only of cardiomyocytes (CM) but also of fibroblasts, pericytes, endothelial, immune cells and others. This increases the complexity of the model but preserves the important heterocellular characteristics of the heart [5]. Myocardial slice culture can serve as an organotypic model for a diseased heart or pathological processes such as remodeling [2,6,7,8], the research of physiological mechanisms [9,10,11] and drug development, specifically drug testing [12,13,14].

Not only cardiovascular diseases, such as heart failure or myocardial infarction, but also the cardiotoxic effects of certain agents or experimental procedures may cause death in cardiac muscle cells. With the goal in mind of preventing or quantifying cardiomyocyte death under these and other conditions, it is essential to reliably and efficiently detect damaged, dying or dead CMs. There are different types of cell death in the heart, such as spontaneous necrosis in acute ischemia or reperfusion injury after myocardial infarction [15,16], as well as regulated apoptosis and necroptosis in cardiomyopathy [17] and reperfusion injury [18,19,20]. Necrosis is commonly referred to as an accidental, uncontrolled form of cell death, morphologically defined by cell swelling, autolysis, irreversible nuclear changes and plasma membrane disintegration or rupture [21,22,23,24]. Since the loss of membrane integrity is a key feature of necrosis, the use of dyes such as ethidium homodimer, propidium iodide and Evans blue, which only penetrate cells with a permeable membrane, is an established method to detect necrotic cells [25,26,27]. Dextran assays are also widely used for this purpose. Dextrans are hydrophilic polysaccharides of varying chain length (3 kDa to 3 MDa) and, due to their chemical properties and size, are excluded from cells with an intact membrane. Thus, dextrans only enter cells in limited amounts through active processes such as endocytosis or, in larger amounts, after the loss of plasma membrane integrity, e.g., rupture of the plasma membrane [28,29]. While the former results in intracellular vesicles filled with dextran, the latter results in the distribution of dextran within the cytosol. When conjugated to a fluorophore, dextran can be used as a fluorescent volume marker to indicate cells with a permeable membrane, i.e., dead cells or cells that are dying. An advantage over other dyes is that fluorophore-conjugated dextrans are available also as chemically fixable variants, which is achieved by the addition of lysine residues. In this way, it is possible to combine a volume marker of dead cells with additional immunofluorescent staining, allowing the differential analysis of living and dead cells within one sample. A drawback, however, is the necessity of an incubation period of several minutes of cells or tissues in an extracellular medium with a high dextran concentration. The incubation itself may affect the experimental outcome. Furthermore, depending on the required volume of the extracellular medium, the assay may be expensive.

The aims of this study were to establish a dextran assay for the detection of dead and viable myocytes in cultured myocardial slices and to find an easy-to-apply alternative to assays based on dextrans or other cell volume markers that does not require the incubation of the tissues with the dye while still alive. The method was required to be inexpensive and to reliably detect viable myocytes in vibratome-cut myocardial tissue slices and—after fixation—allow for combination with immunofluorescent staining. We describe that the immunofluorescent staining of the ryanodine receptor (RyR), the major Ca^2+^ release channel from the sarcoplasmic reticulum (SR), may fulfil these requirements. We show that in cultivated cardiac tissue slices, RyR immunostaining meets these specifications because it disappears in dead or dying myocytes. Moreover, we demonstrate that the number of cells staining positively for RyR correlates with the contractile force and that the method can be used to study structural differences between dead and living myocytes.

## 2. Results

### 2.1. Detection of RyR- and Dextran-Positive Myocytes

**Figure 1:**
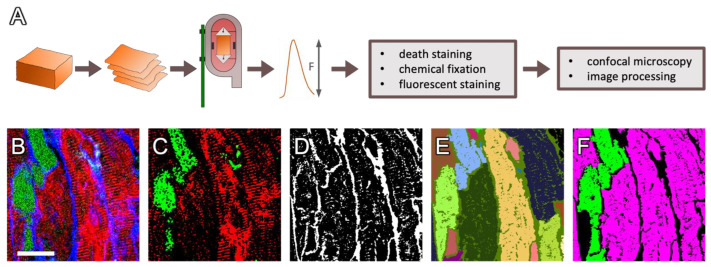
Methodological approach. (**A**) Human and rabbit myocardial slices were created from left-ventricular transmural samples with a vibratome and then cultivated for up to seven days in biomimetic cultivation chambers containing stimulation electrodes and a force transducer. Subsequently, dextran-based death staining was applied, followed by chemical fixation, fluorescent staining, confocal microscopy and image processing. (**B**) Example image of a rabbit cardiac slice stained with fixable, fluorescent dextran (green), for ryanodine receptors (RyR, red) and with wheat germ agglutinin (WGA, blue). (**C**) Histogram-based local thresholds were applied to identify the dextran-positive (green) and RyR-positive (red) pixels, as well as (**D**) the WGA-positive pixels (white). (**E**) Using the WGA distance map, a watershed transform was applied to segment individual myocytes (different colors). (**F**) For each cell segment, the number of RyR- and dextran-positive pixels was counted. If the fraction of these pixels within the segment exceeded a defined threshold, segments were classified as RyR-positive, i.e., living, myocytes (magenta) and dextran-positive, i.e., dead, myocytes (green). Scale bar length in (**B**) is 20 µm and also applies to (**C**–**F**).

Figure 1 shows the magnified subregion of a 1 × 1 mm^2^ confocal microscopic scan of a rabbit myocardial slice that was kept in culture for 24 h and then subjected to the fixable dextran-based death staining assay, chemical fixation and subsequent immunofluorescent staining for RyR clusters. As a marker of the extracellular matrix and cell membranes, wheat germ agglutinin (WGA) was applied. The immunofluorescent staining included permeabilization with Triton-X 100, which, however, preserved the dextran signal (Figure 1B). Thus, it is possible to combine the dextran assay with additional fluorescent markers. Histogram-based thresholds were applied to detect RyR-positive and dextran-positive pixels (Figure 1C) as well as WGA-positive pixels (Figure 1D). Using the Euclidean distance transform of the thresholded WGA signal, cell segments were created, which, in most cases, corresponded to one cell per segment. Smaller errors (overflow or more than one segment per cell) were considered negligible (Figure 1E). Next, the number of dextran- and RyR-positive pixels was counted in each myocyte segment and divided by the total number of pixels within the cell segment. If the resulting fraction exceeded a defined threshold, the segment was classified as an RyR-positive or dextran-positive cell (Figure 1F). Double-positive or double-negative cells were also possible. This segmentation and classification algorithm was applied with equal parameters to all human and rabbit myocytes investigated in this study and was performed without human interaction, i.e., in an automated manner, using custom software based on ITK [30] and Matlab, as previously described [31,32,33].

### 2.2. Dextran and RyR Staining in Rabbit Tissue

#### 2.2.1. Complementary Signal of Dextran and RyR Staining

We acquired 34 two-dimensional tile scans from 22 cultured cardiac rabbit tissue slices obtained from seven New Zealand White rabbits. The culture time ranged from one to seven days. The area of the 2D confocal scans ranged from 0.02 to 2.67 mm^2^.

**Figure 2:**
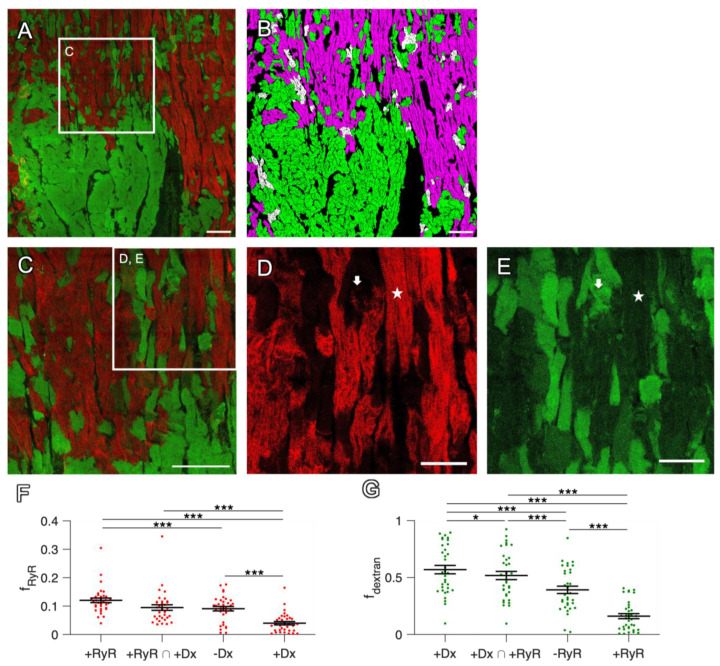
Mutual exclusion of dextran and RyR signals in confocal tile scans of rabbit cardiac slices. (**A**) Overview with complementary dextran (green) and RyR staining (red) of a slice after 7 d in culture. For clarity, the WGA signal is not shown. (**B**) Segmented image of (**A**) with 50.3% of all CM classified as RyR-positive (magenta), 54.2% as dextran-positive (green) and 4.5% double-positive for RyR and dextran (white). (**C**) Magnification of the box in (**A**). (**D**,**E**) Magnifications of the box in (**C**), showing dextran-negative and RyR-positive cells (star) with regularly aligned RyR signal, as well as dextran-positive and RyR-negative cells (arrow). Scale bar lengths: (**A**–**C**) 100 µm, (**D**,**E**) 50 µm. (**F**) The fraction of RyR-positive pixels (f_RyR_) in cell segments that were classified as RyR-positive (+RyR), RyR-positive and dextran-positive (+RyR ∩ +Dx), dextran-negative (−Dx) or dextran-positive (+Dx). (**G**) The fraction of dextran-positive pixels (f_dextran_) in cell segments that were classified as dextran-positive (+Dx), RyR-positive and dextran-positive (+Dx ∩ +RyR), RyR-negative (−RyR) or RyR-positive (+RyR). Data points represent confocal images (n = 35) obtained from 22 slices. Statistical test: paired *t*-test. *p* values after correction for multiple comparisons: * *p* < 0.05, *** *p* < 0.001.

One representative example of a slice cultivated for seven days is displayed in Figure 2. In the overview image (Figure 2A) and the magnification (Figure 2C), cells stained for dextran and RyR can be viewed, with only a marginal overlap of the two signals. When looking more closely at the RyR channel (Figure 2D), there are CMs with a strong RyR signal intensity, while there are others with very low intensity. The first group of myocytes was defined as RyR-positive, if the fraction of RyR-positive pixels after morphological dilation exceeded 15% (see Section 4). These cells typically showed a regularly aligned RyR signal in striated clusters, corresponding to a z-line pattern. In contrast, most cells of the latter group, i.e., RyR-negative myocytes, exhibited no clear signal or a blurry signal without clustering. Figure 2E displays the dextran signal, showing CMs with a homogenous dextran signal distinctive from the background intensity throughout the cytosol. If, after thresholding and dilation, the fraction of dextran-positive pixels exceeded 20%, these cells were classified as dextran-positive myocytes; otherwise, they were classified as dextran-negative myocytes. The pronounced dextran distribution within the cytosol indicates the loss of membrane integrity, which is a characteristic of cell death [29]. Therefore, we identified dextran-positive cells as dead cells and dextran-negative cells as living cells.

The mutual exclusion of RyR and dextran staining was striking (Figure 2). The vast majority of CMs stained positive for either dextran or RyR, while the fractions of CMs staining positive for both (double-positive intersection of both sets) or negative for both was very low (Figure 2B). Quantification and statistical analyses confirmed these observations. The Pearson correlation coefficient between the RyR and dextran signals of the example image in Figure 2 was −0.47, indicating a negative correlation. When analyzing the cell segments detected in all 34 images from 22 slices, dextran-positive (dead) cells exhibited a significantly lower RyR signal density than dextran-negative cells (Figure 2F), while RyR-positive cells contained a significantly lower dextran signal than RyR-negative cells. The fact that RyR-positive cells contained a slightly higher fraction of the RyR signal than dextran-negative cells can be explained by double-negative cells, which may include living myocytes expressing only a few RyR clusters but also non-myocytes—for example, fibroblasts. The statistics of the distribution of thresholded RyR and dextran signals in reference to the total area of all CM segments are also shown in Table 1. Because the tile scan dimensions, i.e., the scanned area, were not equal in all images, we also weighted the size of the image and calculated the total overlap, which was 3.4%, of a total scanned area of 44 mm^2^. For a detailed analysis of each scanned image, we refer to Supplemental Table S1.

In summary, these findings allow us to conclude that dead and dying myocytes lose the RyR signal, suggesting that RyR immunofluorescence is suitable for the detection of viable myocytes in cardiac tissue slices. Conversely, RyR negativity may correspond to the population of dead or dying myocytes.

#### 2.2.2. Artificial Permeabilization of the Membrane with Saponin as a Positive Control

**Figure 3:**
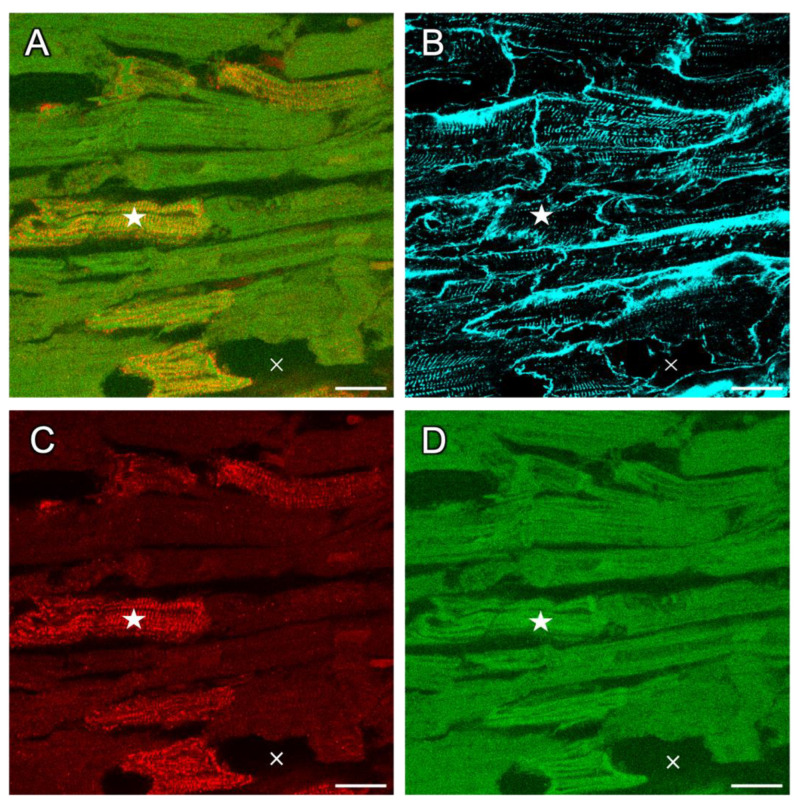
Dextran-RyR co-staining of rabbit tissue permeabilized with saponin. (**A**) Region of a 2D confocal tile scan showing that all cardiac myocytes contain dextran (green), while only some myocytes exhibit RyR immunofluorescent signals (red). The overlay of green and red signals appears as yellow (example marked by a star). (**B**) WGA signal (cyan) showing the extracellular matrix (ECM) and cell membranes. Note that image regions devoid of dextran signal belong the ECM or to areas without any cells (cross). (**C**) RyR signal only. (**D**) Dextran signal only. The cell marked with a star is RyR-positive and displays a regular alignment of RyR clusters despite the uptake of dextran due to the permeabilization of the membrane by saponin. Length of the scale bars is 20 µm.

In our experience, RyR immunofluorescent staining is dependent on the duration of chemical fixation. If fixed with PFA for more than 10–20 min, we consistently observe an attenuated or absent RyR signal, most likely due to epitope masking resulting from excessive protein cross-linking. We therefore investigated whether the absence of the RyR signal in dextran-positive cells could result from the more pronounced or faster chemical fixation of cells with a permeable membrane, because the fixative might obtain quicker access to the cytosol and the SR in these cells. To test this hypothesis, we artificially permeabilized the cell membranes in rabbit myocardial slices with saponin [34,35] while buffering extracellular Ca^2+^ with EGTA to avoid hypercontracture. The slices were then subjected to the same protocol, i.e., incubation in dextran, followed by fixation with PFA and immunofluorescent staining. The result shown in Figure 3 reveals that after saponin treatment, nearly all cells stained positive for dextran, as expected in cells with permeabilized membranes. However, we still observed RyR-positive myocytes with regular cluster alignment. Although many myocytes were RyR-negative, this was not surprising, because, in nearly all cultured slices, we found a mixture of RyR-positive and -negative cells (Figure 2). Analysis of the whole confocal scan of the permeabilized slice (imaged area: 1.2 mm^2^) yielded that 80.1% of RyR-positive cells were also dextran-positive and that 37.4% of dextran-positive cells were also classified positive for RyR. Thus, membrane permeabilization led to abundant dextran staining but did not affect RyR staining. From these results, we conclude that the loss of the RyR signal is not an artefact of the fixation and staining procedure, but rather a result of a biological process occurring during cell death. This implies that RyR immunofluorescent staining may be used to identify living cardiomyocytes in myocardial tissue slices. Moreover, because saponin rendered all cells dextran-positive, the result of this experiment validates the applied dextran assay as appropriate to identify cells with disrupted membrane integrity.

### 2.3. Staining in Human Tissue

**Figure 4:**
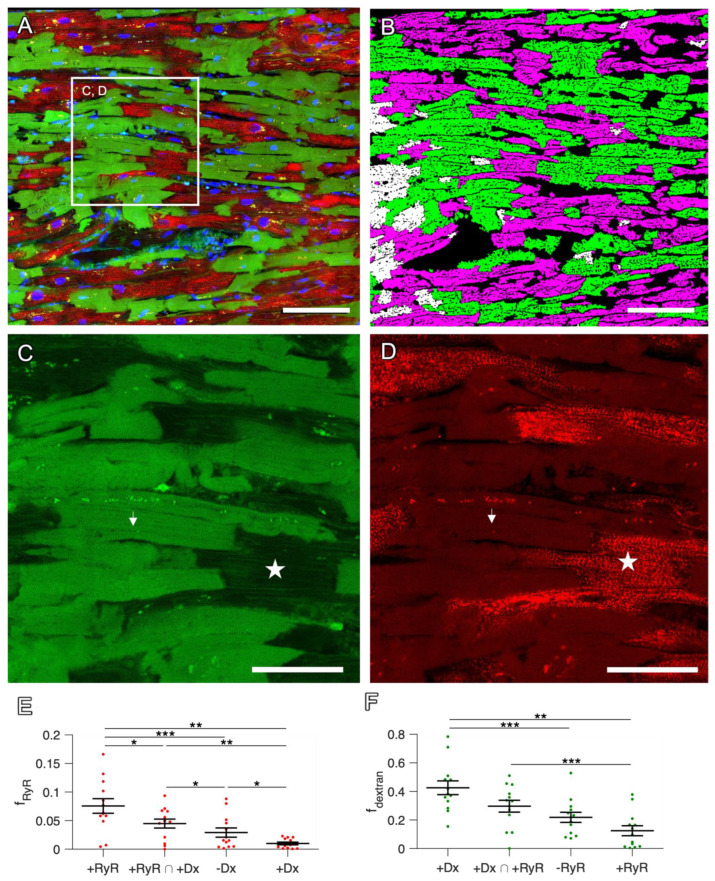
Mutual exclusion of dextran and RyR signals in confocal tile scans of human cardiac slices. (**A**) Overview with signals of dextran (green), RyR staining (red) and DAPI (blue) of a slice after 6 d in culture. For clarity, the WGA signal is not shown. (**B**) Segmented image of (**A**) with 46.0% of all CM classified as RyR-positive (magenta), 45.8% as dextran-positive (green) and 8.6% double-positive for RyR and dextran (white). (**C**,**D**) Magnifications of the box in (**A**), showing dextran-negative and RyR-positive cells (star) with regularly aligned RyR signal, as well as dextran-positive and RyR-negative cells (arrow). Scale bar lengths: (**A**,**B**) 100 µm, (**C**,**D**) 50 µm. (**E**) The fraction of RyR-positive pixels (f_RyR_) in cell segments that were classified as RyR-positive (+RyR), RyR-positive and dextran-positive (+RyR ∩ +Dx), dextran-negative (−Dx) or dextran-positive (+Dx). (**F**) The fraction of dextran-positive pixels (f_dextran_) in cell segments that were classified as dextran-positive (+Dx), RyR-positive and dextran-positive (+Dx ∩ +RyR), RyR-negative (−RyR) or RyR-positive (+RyR). Data points represent confocal images (n = 13) obtained from 4 slices. Statistical test: paired *t*-test. *p* values after correction for multiple comparisons: * *p* < 0.05, ** *p* < 0.01, *** *p* < 0.001.

Since the long-term organotypic cultivation of cardiac slices is commonly used for human heart samples, which is of great interest in cardiac research [1,4,8], we next sought to verify whether our findings obtained from rabbit hearts could be translated to human tissues. For this purpose, we cultivated four slices from two left-ventricular samples obtained from human failing hearts for one to six days and recorded 13 confocal microscopic scans covering a total area of 6.5 mm^2^. It is evident from Figure 4 that—very similarly to rabbit—the cultivated human tissue slices contained predominantly CMs that stained positive for either RyR or dextran. Only a few cells were double-positive (Figure 4B–D). This was confirmed by a statistical analysis of all images, showing that the RyR signal density in dextran-positive dead or dying cells was significantly lower than in dextran-negative cells (Figure 4E and Table 2). Conversely, the dextran signal density was lowest in RyR-positive, i.e., living, cells (Figure 4F). Moreover, in human cells, the overlap in the total area scanned (1.2%) was low.

In summary, this indicates that the presented dextran assay is applicable to human cardiac slices as well and that the RyR signal can be used to detect the majority of living cells. Inversely, a lack of RyR signal suggests that a myocyte is dead.

### 2.4. Correlation with Functional Data

**Figure 5:**
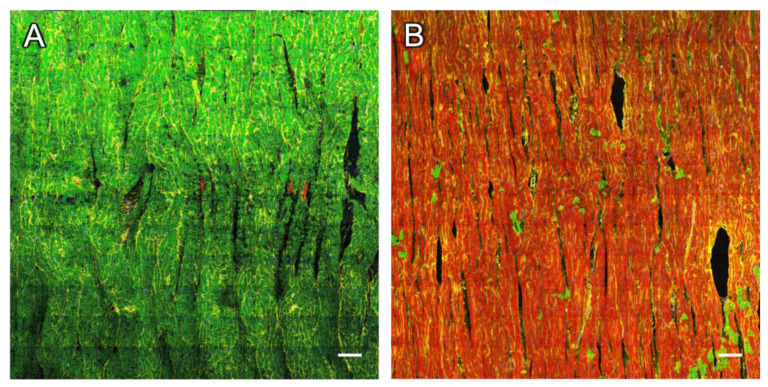

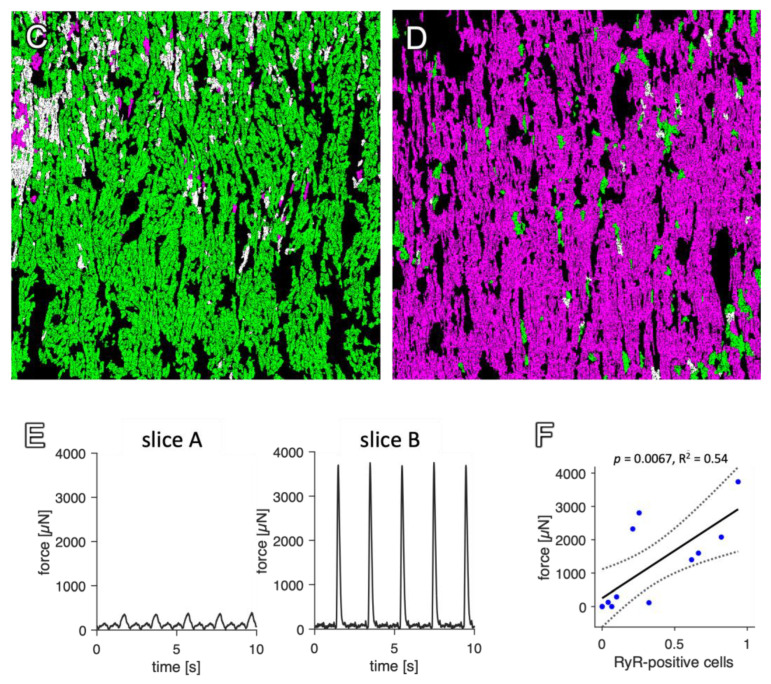
Correlation of RyR-positive myocytes with contraction amplitude in rabbit slices after 1d in culture. (**A**) 2D confocal tile scan image of a tissue slice with low contraction force. Dextran signal (green) and RyR signal (red) are shown. Overlay appears in yellow. (**B**) Image of a slice with higher contraction force than (**A**). (**C**,**D**) Segmented images of (**A**,**B**), respectively, classified into RyR-positive (living, magenta) and dextran-positive (dead, green) cells. Double-positive cells appear white. Scale bar length is 100 µm. (**E**) Example traces of contraction force measurements corresponding to the slices shown in (**A**,**B**). Pacing frequency was 0.5 Hz. (**F**) Linear regression model with contraction force as dependent variable and fraction of RyR-positive cells as predicting variable (n = 12 observations). F-statistic vs. constant model: *p* = 0.0067; coefficient of determination: R^2^ = 0.54.

The fraction of viable myocytes, their alignment and their overall condition or internal biological state is crucial for the effective contraction of the slice. After identifying dextran as a suitable marker for dead and RyR for living myocytes in cultured cardiac tissue slices, we asked whether the fraction of living CMs within the analyzed tissue had implications on the functional data. We used culture chambers in which myocardial tissue slices contracted under continuous electrical stimulation. During the culture, the contraction force of each beat was measured as the amplitude of the spring wire deflection multiplied by the spring constant [1]. Figure 5A shows a 1.5 × 1.5 mm^2^ confocal tile scan of a rabbit slice with a low contraction force of 290 µN (Figure 5E, left graph). The scanned area consisted of 10.0% RyR-positive (living) CMs. Figure 5B depicts a slice with an amplitude of 3793 µN (Figure 5E, right graph) and a fraction of 94% living CMs within all detected myocytes in the scanned area. We then investigated the correlation of the fraction of living CMs from 12 rabbit slices after 24 h in culture with their contraction force in a linear regression model. Regions were randomly chosen, and the researchers were blinded against the contraction forces. Although the scanned volumes were small in comparison to the slice volume, we obtained a distinct positive correlation (*p* = 0.006 vs. constant model, R^2^ = 0.54), as displayed in Figure 5F. This substantiates the relevance of the live–dead staining applied here by suggesting that only RyR-positive cells contribute to the force generation of the slice and fits well with the presumption that RyR-negative cells are either dead or not able to produce a sufficient Ca^2+^ signal required for active contraction.

### 2.5. Using RyR and Dextran to Compare Living and Dead Cells

**Figure 6:**
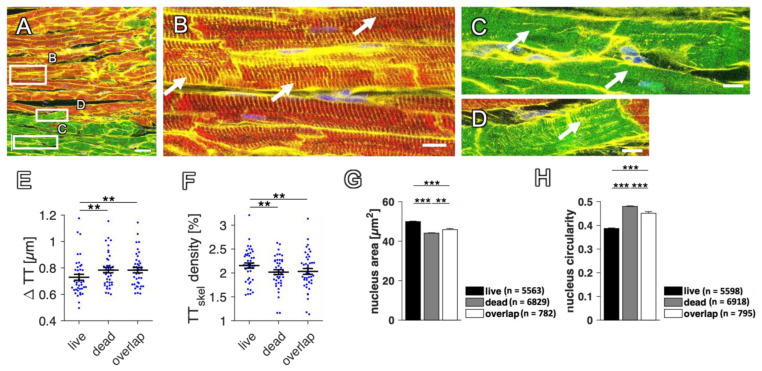
Analysis and comparison of microstructural parameters in living and dead rabbit cardiomyocytes. (**A**) WGA (yellow), RyR (red), dextran (green) and DAPI (blue) signals in a confocal scan of a rabbit slice after 1 day in culture. (**B**) Magnified region indicated in (**A**) with RyR-positive (living) myocytes. Arrows point to t-tubules with high density and regularity. (**C**,**D**) Magnified regions indicated in (**A**) with dextran-positive (dead) myocytes. (**C**) Arrows point to remodeled t-tubules and regions with low t-tubule density and regularity. (**D**) Arrow points to dense t-system. Scale bar length in (**A**) is 50 µm. Scale bar length in (**B–D**) is 10 µm. (**E**) Mean intracellular t-tubule distance (ΔTT) was analyzed in living (live), dead and double-positive (overlap) cardiomyocytes. Note that higher values indicate fewer t-tubules. (**F**) Density of the skeletonized (to one-pixel width reduced) t-tubules in living, dead and double-positive cardiomyocytes. n = 62 images, 35 slices, N = 8 animals, paired *t*-test. *p* values after multiple comparison correction: ** *p* < 0.01. (**G**) Mean nucleus area in the different myocyte compartments; n indicates the number of analyzed nuclei. (**H**) Circularity of the nuclei, calculated by dividing the short axis by the long axis of each nucleus. *p* values after multiple comparison correction: ** *p* < 0.01, *** *p* < 0.001, unpaired *t*-test.

Apart from quantifying the fraction of viable and dead myocytes in a cardiac tissue slice, this method allows the comparison of structural parameters within the subpopulations of living and dead CMs. Dead myocytes, staining positive for dextran, are sometimes hypercontracted and structurally altered, but sometimes appear healthy apart from the loss of RyR. For example, they sometimes still possess a dense transverse tubular system (t-system), a network of membrane tubules found in reaching into the cytosol, which is important for coupling membrane-bound L-type Ca^2+^ channels to RyRs in adult CM [36]. In Figure 6, the t-system is shown to be dense in living myocytes (Figure 6B) but heterogeneous in dextran-positive cells. While some dead CMs display t-system loss (Figure 6C), others still contain a number of regular t-tubules, as evident from WGA staining (Figure 6D).

To compare the t-system between RyR-positive (live), double-positive (overlap) and dextran-positive (dead) myocytes, the mean distance to the closest transverse tubule (∆TT) and the t-tubule skeleton density were calculated within the respective subpopulations, using the WGA signal [31,37]. In a total of 35 slices after 1 d to 7 d in culture from eight rabbit hearts, the mean distance of TT in living CMs was significantly higher than in dead CMs and the skeleton density of TT was significantly lower (Figure 6E,F). Nevertheless, the difference between the groups was small (∆TT = 0.75 ± 0.02 µm live, 0.79 ± 0.01 µm dead). Surprisingly, this suggests only minor effects on the t-system during cell death in cultivated cardiac slices.

Another structural change that is expected and can be compared here in dead and living myocytes concerns the nucleus. During cell death, the nucleus may change its size and shape [21]. Thus, because all slices were co-stained with DAPI, we evaluated the mean nuclear area and circularity (Figure 6G,H). The nucleus area was slightly, but significantly, larger in living than in dead CMs (49.4 ± 0.2 vs. 44.0 ± 0.2 µm^2^), while double-positive cells (overlap) were closer to dead CMs (45 ± 0.2 µm^2^). The circularity, however, was higher in dead than in living CMs. These changes in area and circularity are surrogates of karyopyknosis and karyorrhexis associated with cell death [21].

These results demonstrate that the presented method can be applied to investigate microstructural differences between living and dead or dying myocytes.

### 2.6. Use of RyR Immunofluorescence without Dextran to Detect Viable CMs

Although our results show that fixable fluorophore-conjugated dextran works well in myocardial slices, it remains relatively expensive and takes up a channel for imaging, which could be used to analyze other relevant structures, and the assay is time-consuming and must applied before fixation. The additional incubation period could alter or confound the results of experiments in living tissue. Therefore, although the dextran assay is a sensitive and well-accepted method to detect dead myocytes, an alternative without these drawbacks is desirable. Considering the apparent specificity of RyR immunofluorescence for viable CMs shown here, it seems reasonable to use RyR staining as a sole marker.

**Figure 7:**
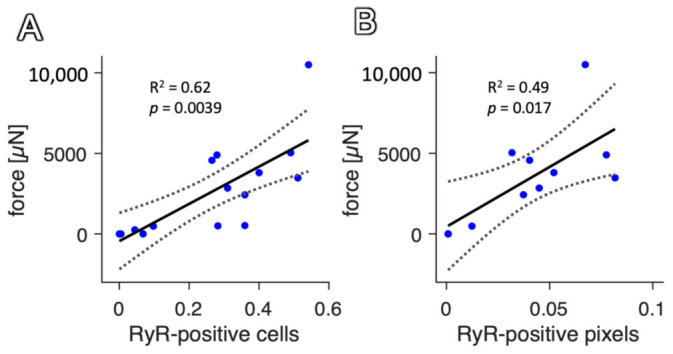
Correlation of contraction force with RyR-positive myocytes and pixels in rabbit tissue slices stained solely for RyR. (**A**) Linear regression model with contraction force as dependent variable and fraction of RyR-positive cells as predicting variable (N = 11). F-statistic vs. constant model: *p* = 0.0067; coefficient of determination: R^2^ = 0.54. (**B**) Linear regression model with contraction force as dependent variable and fraction of RyR-positive pixels as predicting variable (N = 11). F-statistic vs. constant model: *p* = 0.017; coefficient of determination: R^2^ = 0.49.

In Figure 7A, we show the correlation of RyR-positive myocytes with the contraction force in rabbit slices that were stained without dextran, but still for RyR and with DAPI and WGA, after 1 to 7 days in culture. The image processing was identical to that for double-stained slices (Figure S1). As expected, we found a clear and significant correlation (R^2^ = 0.62, *p* < 0.01) between the fraction of living myocytes and contraction force, confirming the results from Figure 5F in a different population of slices. Because the identification of cell segments is rather complicated, we next investigated the correlation of RyR-positive pixels with the contraction force (Figure 7B). Again, we found a positive correlation and it was found that the predictive value was only slightly lower (R^2^ = 0.49, *p* < 0.05).

This suggests that the dextran staining can be omitted in the laboratory routine and RyR staining, a very commonly used staining method in basic cardiac research, can be used as a marker for viable cells and is even able to predict the functional parameters of the slices, particularly the contraction force. If dead cells are of interest, RyR-negative cells can be studied. An additional conclusion that can be drawn from the high similarity of the results in the populations of RyR-only stained slices and those stained also with dextran is that the dextran assay had no confounding effect on the specificity of RyR staining for viable CMs. In other words, the loss of RyR staining was not a result of dextran entering the cells.

### 2.7. RyR–Dextran Co-Staining in Fresh Cardiac Slices

Cardiac tissue slices are produced with a vibratome from blocks of living tissue of explanted animal or human hearts while being constantly immersed in cold buffer solution. CMs can be damaged during the slicing, but also earlier during tissue procurement or transport. Quantifying the degree of damage after slicing could therefore provide useful feedback when trying to improve these processes. Thus far, it has been demonstrated only in slices that were cultivated for at least 24 h that RyR staining can replace the dextran assay to distuinguish between viable and dead myocytes. Therefore, we sought to verify the presented method in freshly cut, non-cultivated slices. Moreover, we assessed whether significant myocyte damage could be observed already before culture, i.e., before re-warming and re-oxygenation, because the biological processes that cause the disappearance of RyR staining might run more slowly at cold temperatures or only occur as a result of reperfusion-like injury when installing the slices in culture.

**Figure 8:**
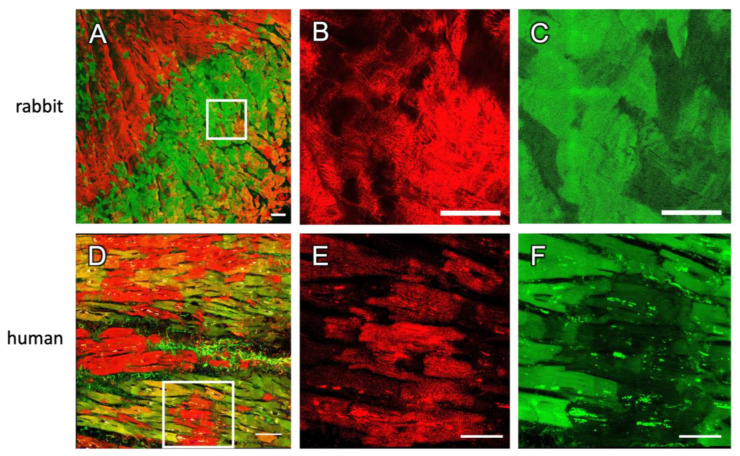
Two-dimensional tile scans of non-cultivated rabbit and human tissue slices after cutting with a vibratome. The tissue was stained directly after slicing with dextran (green) and for RyRs (red). (**A**–**C**) Rabbit slices with magnifications of the indicated region in (**A**) shown in (**B**,**C**). (**D–F**) Human slices with magnifications of the indicated region in (**D**) shown in (**E**,**F**). Scale bar length: (**A**,**D**) 100 µm, (**B**,**C**,**E**,**F**) 50 µm.

To this aim, we stained fresh rabbit and human tissue slices directly after sectioning with the vibratome and investigated the overlap of the RyR and dextran signals. Figure 8A–C show images of stained rabbit tissue and Figure 8E,F of human tissue. Note that regions containing large amounts of dextran-positive cells were chosen to demonstrate double-positive cells. Similarly to cultivated slices, we observed that most cells were only positive for RyR or only positive for dextran, but the two signals appeared to overlap more than in cultured slices. This means that there was a larger number of double-positive myocytes. We also found that the fraction of double-positive CMs was lower in fresh human than in fresh rabbit slices (rabbit: 16.8 ± 5.1%, n = 3 slices, human: 5.9 ± 1.9%, n = 4 slices). Looking at the magnifications (Figure 8B,E), one can see that cells stained positive for dextran often had a weaker but not completely vanished RyR signal, leaving a blurry background-like signal, similarly as described in cultured tissues. These findings suggest that the co-staining of dextran and RyR is possible and useful also in non-cultured tissue sections and could be used to detect damage from tissue handling and cutting with the vibratome. However, the RyR staining may not be as specific for living (dextran-negative) myocytes as in uncultured slices.

### 2.8. Accuracy of RyR Immunofluorescence as a Test for Viable Myocytes

Considering the dextran assay as an established test for the detection of dead cells with presumably high sensitivity and specificity (“gold standard”), it is possible to define the results from dextran staining as truth. Dextran-positive myocytes are dead; dextran-negative myocytes are viable. The RyR immunofluorescent signal tests for living myocytes (positive test result), and a lack of RyR signal would indicate dead myocytes (negative test result). These results can be compared to the presumed truth to obtain the sensitivity and specificity, as well as the positive and negative predictive values. Table 3 shows these characterisitcs in the different groups. The specificity was highest in cultured slices, fitting with the observation of higher numbers of double-positive cells. The confusion matrices for all groups can be retrieved from Table S2.

Note that the classification of cell segments was based on the fraction of RyR-positive pixels within each segment. If this fraction was higher than the chosen threshold, the segment was classified as positive. It is possible to alter the threshold to obtain higher values for sensitivity or specificity, whichever is more important. An example is displayed in Figure S2, showing the receiver operating characteristics (ROC) as well as the dependence of the true and false positive rates on the selected threshold.

### 2.9. Changes in RyR and SERCA Regularity in Dying Myocytes 

**Figure 9:**
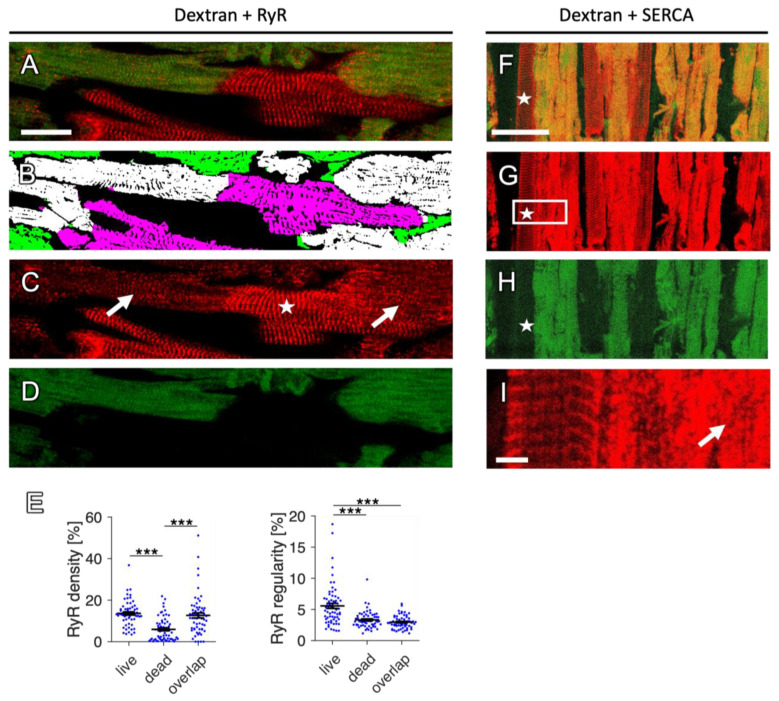
RyR and SERCA patterns in dextran-stained rabbit cardiomyocytes. (**A**) RyR (red) and dextran signal (green) shown as overlay. (**B**) Segmented image with myocytes classified as dextran-positive (green), RyR-positive (magenta) or double-positive (white). (**C**) RyR signal only, showing a regular z-line pattern in dextran-negative myocytes (star) and a blurred, less clustered pattern in double-positive myocytes (arrows). (**D**) Dextran signal only. Scale bar length in (**A**) is 20 µm and also applies to (**B**–**D**). (**E**) Quantitative analysis of RyR density (percentage of RyR-positive pixels within myocytes) and regularity, measured by the percentage of the image energy explained by the spectral density in the frequency domain 1/(2.5 µm) through 1/(1.5 µm). These parameters were calculated in RyR-positive (live), dextran-positive (dead) and double-positive (overlap) myocytes. *p* values after multiple comparison correction: *** *p* < 0.001 (paired *t*-test, n = 62 images from 35 slices, N = 8 rabbit hearts). (**F**) Overlay of SERCA (red) and dextran (green) signals showing dextran-positive cells that also contain SERCA signal (appearing yellow). A dextran-negative cell with regularly aligned structure of the SERCA signal (z-line pattern) is highlighted (star), while, in dextran-positive cells, the SERCA pattern is irregular. A larger image region is provided in the Supplementary Materials (Figure S2). (**G**) SERCA signal only. (**H**) Dextran signal only. (**I**) Magnification of the region indicated in (**G**). The arrow points to a dextran-positive cell with loss of the regular SERCA distribution. Scale bar length in (**F**) is 50 µm and also applies to (**G**,**H**). Scale bar length in (**I**) is 5 µm.

A relatively small population of the myocytes stained positive for dextran and RyR and the degree of this overlap of the RyR and dextran signals was higher in fresh than in cultivated slices. It is conceivable that double-positive cells are in an intermediate state where the cell membrane is already permeable but RyR staining has not yet been lost. In fact, presuming that both processes, i.e., the loss of membrane integrity and alterations that lead to the loss of RyR staining, occur gradually over a certain amount of time, one would expect such an intermediate state. One possible explanation for the loss of the RyR signal is the degradation of the SR. In Figure 9, we show a close-up image of a region from a rabbit slice after one day in culture, containing myocytes that are exclusively RyR-positive or double-positive. Close inspection of the RyR signal in the dextran-negative CMs reveals the well-known regular pattern that matches the z-line alignment of the sarcomeres. However, in double-positive cells, RyR clusters appear unorganized, as irregular points or blurred (Figure 9C), although they still show relatively high intensity. We confirmed these observations quantitatively by calculating the RyR density and regularity in the respective myocyte subpopulations (Figure 9E). The RyR density, expressed as the percentage of pixels above the background, did not differ in the overlap (double-positive) cells when compared with living cells, but was significantly higher than in dead (exclusively dextran-positive) cells. In contrast, the RyR regularity, calculated from the power spectral density in the frequency domain, was as low in the overlap as in dead cells. This suggests that the location of RyR clusters becomes irregular before RyR staining disappears.

If the changes observed in the RyR distribution are a result of SR degradation, one would expect an altered pattern also of other proteins located in the SR membrane. We therefore stained the sarcoplasmic/endoplasmic reticulum calcium ATPase (SERCA), a transporter that is expressed abundantly in the SR of cardiac myocytes (Figure 9F–I). We found that the SERCA staining was regular in dextran-negative cells, resembling a z-line pattern, but strikingly blurred and planar in dextran-positive cells. A larger image of the SERCA-stained slice can be viewed in Figure S3.

Collectively, our data suggest that some form of SR membrane degradation may underlie the loss of RyR staining signals in dead and dying cells, which also fits with the observation that more double-positive stained myocytes exist in uncultured, permanently cooled cardiac slices, where these degradation processes may occur more slowly.

## 3. Discussion

In this study, we utilized an assay for the detection of dead cardiomyocytes that is based on the fundamental biology of cells losing their membrane integrity during cell death, especially necrosis, to establish and validate a new, simpler method that can differentiate viable from dead myocytes in rabbit and human cardiac tissue slices cultivated in biomimetic chambers. Further, we correlated the assay with functional parameters and demonstrated that it allows us to compare structures, such as the t-system and the nucleus, between live and dead myocytes. Finally, our findings provide an approach to studying cell death and related mechanisms in future studies.

### 3.1. RyR–Dextran Assay as a Suitable Live–Dead Staining Method in Cultured Cardiac Tissue Slices

Dyes that only penetrate cells with a permeable membrane are commonly used to detect dead cells that have lost their membrane integrity [25,26,27]. Here, we used fixable dextran conjugated to fluorophores in an assay based on this principle, defining it as the gold standard for the validation of RyR immunofluorescence (IF) as a novel marker for viable myocytes. In cultivated human and rabbit slices, we observed the mutual exclusion of the RyR and dextran signals, resulting in an only small overlap containing double-positive myocytes (Figure 2 and Figure 4, Table 1 and Table 2). Importantly, the mean RyR signal intensity in dextran-negative (=viable) cells was significantly higher than in dextran-positive (=dead) cells, allowing us to set a threshold to distinguish between these two cell populations (Figure S2).

It is important to note that the relatively high fractions of dead cells in our images were partly a result of slice selection and must not be considered representative of human or rabbit fresh or cultivated slices. Our goal was to present and validate the method and not to compare the viability of tissues in different species or under different culture conditions. However, the researchers were blinded against the slice groups and functional data during image acquisition, allowing an unbiased investigation of the correlation between the contraction force and myocyte viability (Figure 5F and Figure 7).

Based on our findings, we suggest that this method can be used to evaluate the fraction of living CMs as a surrogate for cell survival and function, which could—similarly to the contraction force—be useful in toxicity screening tests or to evaluate experimental groups. Because most slices are heterogeneous in myocyte orientation, tissue composition or injuries from tissue procurement, slicing and culture, an area or volume large enough to be considered representative should be scanned with the microscope, ideally from different regions of the tissue. Another aspect to consider when using this method is to confirm that the staining generally works, because there are several explanations as to why IF staining fails and one cannot readily assume that all CMs are RyR-negative and thus dead if there is a complete lack of signal. As with all types of laboratory assays, technical errors may lead to false results. Here, our recommendation is to check whether at least some cells exhibit a clear RyR signal or to include viable tissue as a positive control to avoid falsely classifying cells as dead. 

Our automated workflow of segmenting images, detecting myocytes and classifying them into live and dead requires elaborate automated image analysis techniques. While this allows for a high throughput and unbiased analysis, it may be difficult to establish in other laboratories. As a straightforward way to use RyR staining with default image analysis software, such as ImageJ, we suggest the following workflow. Inspect the image and identify cardiomyocytes of high and low signal intensity of the RyR staining. Set an intensity threshold that divides the signal into the background (myocyte with low or no RyR staining) and signal (myocyte with high RyR staining). After application of the threshold, manually select the region of interest for analysis. This could be the whole image or a subregion of the tissue or a single myocyte. By counting the RyR-positive pixels and dividing this number by the total number of pixels within the region of interest, it is possible to compare cells or differently treated tissues with each other.

### 3.2. RyR–Dextran Assay in Non-Cultured Cardiac Tissue Slices

In fresh tissue, we observed a greater overlap among the RyR and dextran signals. Because dextran indicates dead whereas RyR indicates living cells, this means that, under the presumption of dextran as a gold standard, the false positive rate of the RyR-based viability detection was higher, resulting in lower specificity in non-cultivated than in cultivated tissue slices. We therefore would not recommend without reservation the use of RyR staining as a sole marker in freshly cut myocardial slices. As discussed below, a possible reason that RyR immunofluorescence is less specific in these slices is that the biological processes causing the disappearance of the staining take more time at cold conditions and are not yet completed. However, the non-specificity of the dextran test in fresh slices is a possibility that should also be considered. As described in Fisher 2019 [1] and routinely observed in our laboratory, cardiac slices show transient but mild hypercontracture for 10–20 min immediately after the installation and rewarming of the culture chambers. This could indicate reversible myocyte damage and, possibly, the less pronounced permeability of the cell membrane that can be repaired. Thus, although dextran can enter the cytosol, it may not necessarily mean that all dextran-positive cells are irreversibly damaged and will die. Some of them may have the potential to recover. This idea also fits with the common observation that, after enzymatic cardiomyocyte isolation, the extracellular Ca^2+^ needs to be increased slowly to avoid the hypercontracture and death of the myocytes, indicating reversible membrane damage. However, additional studies are required to answer these questions and to validate the dextran assay in freshly cut cardiac slices stored under cold conditions.

An interesting question is whether the shown method can detect necrotic cells in tissues that were fixed prior to sectioning and staining. In many of our previous studies, e.g., [31], we did not observe patterns of single myocytes or groups of myocytes with and without RyR staining in tissue slices created after fixation. It seems reasonable to assume that healthy rabbit hearts do not contain significant amounts of dead myocytes. Based on published studies, we believe that this is also true for the human, chronically failing myocardium [38]. In acute myocardial infarction or ischemic heart disease, there might be larger numbers of necrotic cells [15], but these conditions were not investigated here. Thus, the cell death observed in fresh (non-cultured) slices may have happened mainly during tissue transport, storage and slicing on the vibratome. In cultured slices, which lack an intact immune system, the removal of dead cells may occur much more slowly than in vivo. 

One should also keep in mind that the dextran assay can only detect cell death accompanied by the disintegration of the membrane, such as necrosis and necrosis-associated forms [39]. In apoptotic cells, on the other hand, the cell membrane remains intact [40], suggesting that apoptotic cells are negative for dextran. However, we have not evaluated the sensitivity of the dextran assay for apoptotic cells and did not investigate the effects of apoptosis on RyR staining. In heart disease, apoptosis is a contributing form of cell death but overall still a rare event, while, in non-failing hearts, cardiomyocyte apoptosis is almost non-existent [17,38]. Therefore, the presented approach identifies the main type of myocardial cell death, which is necrosis.

### 3.3. Use of This Method to Compare the Structures of Viable and Dead Myocytes

Another application is the comparison of living and dead (or dying) myocytes. Here, we demonstrated differences in nucleus morphology and t-system density. Detecting the nucleus and investigating its morphology is of interest not only when studying cell death [21], but also in viable cells when studying, for example, ploidy or the cell cycle and turnover [41]. With the presented methods, it would be possible to identify nuclei from dead and living myocytes, using dextran or RyR as their respective markers, as demonstrated in Figure 6.

Surprisingly, the mean t-system density was only slightly reduced in dead myocytes in cultivated cardiac slices (Figure 6E,F), although some cells exhibited severe t-system loss (Figure 6C). This could indicate that t-system loss is not a typical event during myocyte loss, but that active processes of living myocytes are required for t-system loss and remodeling as it is present in human heart failure [31]. However, cell death in CMs is commonly accompanied by the hypercontraction and shrinkage of the cytoplasm [42,43,44], because Ca^2+^ enters the cell, causing sarcomeres and the cytoskeleton to contract. This alters the cell geometry and most likely influences the structure and density of the t-system. From hypercontracture and volume loss, one would expect a decrease rather than an increase in t-system density, which may have counteracted the possible loss of t-tubules during cell death and may be another reason for the only slightly lower values of mean t-system density in dead cardiomyocytes. It is also possible that t-system loss or remodeling requires factors that are not present the in vitro model of myocardial culture—for example, inflammatory processes and the activation of immune cells.

### 3.4. RyR as a Myocyte Marker

Often, it is not the comparison of living versus dead myocytes that is of interest, but one is interested in analyzing the effects of experimental procedures or treatments on viable CMs or to distinguish myocytes from other cell types [45]. In this case, dead myocytes or non-myocytes could even confound the results. By setting a relatively high threshold for the classification of cells as RyR-positive, one can achieve high specificity and omit dead myocytes or non-myocytes from the analysis. As an alternative to other myocyte-specific immunostainings, e.g., against alpha-actinin, myosin or troponin T, we therefore suggest to use RyR as a viable myocyte marker, as already done in some of our previous studies [2,31].

### 3.5. Interrelation of the Staining Result with Functional Parameters

We showed a positive correlation of living myocytes with the contractile force of the cardiac tissue slices. This is a very logical finding in several aspects. First, assuming that most RyR-negative myocytes are dead and vice versa, one would expect a larger amount of actively contracting viable myocytes contributing to the force of the whole tissue slice. Second, ryanodine receptors are of particularly high importance for calcium-induced calcium release and, thus, for excitation–contraction coupling in cardiac myocytes. Even if a myocyte is still alive but has no or only small amounts of RyR clusters left, the Ca^2+^ signal would be very weak, resulting in poor contraction.

On the other hand, approximately 50% of the observed variability in the contraction force was not explained by the amount of viable myocytes (Figure 7). This is not surprising because a variety of other causes exist for differing contraction forces, such as the level of fibrosis, orientation of cardiomyocytes within the slices, time in culture, size dimensions of the tissue slice as well as sample–sample or heart–heart variations and others.

Nevertheless, the presented correlation is not only an example of an application where we used representative images of the tissue as a marker of viability to predict function, but also affirms this method. Furthermore, it shows that besides using the fraction of living myocytes, requiring the segmentation of the tissue, the simple fraction of RyR-positive pixels is already a good predictor (Figure 7B) of slice contractile function.

### 3.6. Presence and Influence of Non-Myocytes in the Culture System and In Vivo

An advantage of the cultivation of beating myocardial slices is the preserved heterocellularity. While RyRs are specific for myocytes and, thus, cannot be used to classify or evaluate non-myocytes, the presented dextran assay may also work to investigate cell death in other cell types—for example, fibroblasts or endothelial cells. When looking closely at Figure 1B,C, dextran staining can be observed between myocytes, overlapping partly with the WGA signal. This is the typical location of fibroblasts within the extracellular matrix. We observed small numbers of dextran-positive non-myocytes in several images. However, here, we focused on myocytes. Using a specific marker for non-myocytes—for example, immunofluorescent staining against vimentin—in conjunction with fixable dextran, it would be possible to quantify the fraction of dead non-myocytes.

Another aspect related to non-myocytes is that even in slices cultivated for 7 days, we found relatively high fractions of dead myocytes in some slices. It is unlikely that these myocytes had died acutely because this would have resulted in a sudden reduction in contractile force, which we did not observe. Instead, it is likely that these myocytes had been dead for several days. In vivo, such observations are uncommon because necrotic cell death triggers immune cells and causes inflammation, which eventually leads to the removal of dead cells and replacement by fibrosis [46]. In this in vitro model, however, an effective immune system is missing. This possibly leads to the deficient removal of dead cells in the cultured slices. This may also explain why this pattern of RyR-positive and -negative CMs cannot be seen in directly fixed fresh myocardium [31,47].

### 3.7. Degradation of the SR as a Suggested Mechanism of RyR Staining Loss 

Given the importance of RyR channels for cardiac excitation–contraction coupling, the immunofluorescent imaging of RyRs is very frequently used in cardiac research [31,37,48,49]. In a physiological state, RyRs are organized in clusters within the SR and regularly aligned with z-lines and t-tubules [50,51]. In cardiac diseases, such as heart failure and myocardial infarction, the dispersion of RyR clusters [49,52] and the remodeling of SR proteins close to t-tubules has been reported [31,51,53,54]. A loss of volume or the collapse of the SR has also been shown to be relevant in cardiac diseases [48,55,56] and some authors even describe phospholipid depletion during cell death, affecting intracellular membranes as the membrane of the SR in cardiomyocytes [57,58,59,60]. Our study fits well with these reports, providing evidence that the processes altering the SR structure and/or integrity are involved (Figure 9). We suggest that SR degradation is an early event during cardiomyocyte death, because we observed altered and irregular RyR cluster patterns specifically in dextran-positive myocytes. The fact that the two assays indicate two different aspects of cell death may also explain why their results are not fully equivalent: while the dextran staining visualizes cell membrane disintegrity, the lack of RyR immunofluorescence may exhibit intracellular organelle degradation. Further investigation of these mechanisms should be the subject of future research.

## 4. Materials and Methods

### 4.1. Cardiac Samples

The use of rabbits for this study was approved by the Animal Care and Use Committee Mittelfranken, Bavaria, Germany. Female New Zealand White rabbits (2.5–3.0 kg) were purchased at Charles River Germany. Animals were sedated with ketamine/xylazine i.m. (25 mg/kg and 5 mg/kg) and killed by i.v. injection of pentobarbital (200 mg/kg). Immediately afterwards, the heart was removed.

Human tissue samples were obtained from the Cardiac Transplant Program (University of Utah Health Science Center). The institutional review board of the institution approved the study, and all patients provided informed consent.

### 4.2. Tissue Preparation and Cultivation

After excision, rabbit hearts were immediately immersed in cold cardioplegic solution (containing, in mmol/L, potassium glutamate 80, NaCl 10, 2,3-butanedione monoxime 30, sucrose 50, KH_2_PO_4_ 25, MgSO_4_ 5, CaCl_2_ 1, allopurinol 1, adenosin 5, glutathione 5, pH 7.4) and transported to the laboratory. The epicardial fat, apex, right ventricle and septum were removed and the left ventricle (LV) was cut into 2–4 tissue blocks used for slicing with a vibratome (VT1200S, Leica, Wetzlar, Germany) and subsequent tissue culture, as described [1]. Similarly, human left-ventricular samples were transported, stored, cut into blocks of approximately 1 cm × 1 cm, embedded into low-melt agarose and cut into slices of 300 µm thickness and installed in biomimetic cultivation chambers (MyoDish 1.0, InVitroSys, Gräfelfing, Germany), according to published protocols [1,2,10]. Tissue slices were cultured for at least 24 h and up to one week. As a culture medium, we used M199 (Sigma M4530), supplemented with insulin (10 ng/mL), transferrin (5.5 μg/mL), selenium (6.7 ng/μL), β-mercaptoethanol (50 μM), penicillin (100 units/mL)/streptomycin (0.1 mg/mL). All slices were slightly stretched to a diastolic preload of 1000 to 1500 µN and paced at 0.5 Hz. Two thirds of the medium was exchanged every 48 h. 

### 4.3. Dextran Assay and Immunostaining

Dextran 3 kDa conjugated to FITC (Thermo Fisher, D3306) and dextran 10 kDa conjugated to AF-488 (D22910, Thermo Fisher, Braunschweig, Germany) were used for the staining. Remaining in the chambers, cultivated slices were incubated with dextran (2 mg/mL) dissolved in 0.5 mL of M199 for 10 to 20 min. Before dextran incubation, the electrodes were removed and the chambers placed back in the incubator at 37 °C and 5% CO_2_ under continuous rocking. For the positive control, fresh tissue was incubated with EGTA + saponin (10 mM + 50 μg/mL) for 10 min and placed back into the incubator before being incubated with dextran. Fresh slices were incubated with dextran (2 mg/mL) in cutting solution [1] at room temperature (RT). The dextran medium was removed and the tissue immediately fixed with 2% paraformaldehyde (PFA) in phosphate-buffered saline (PBS) for 10 min and washed three times with PBS for 5 min each. 

For immunostaining, the slices were incubated with primary antibody against cardiac ryanodine receptor (IgG1, mouse, C3-33, Thermo Fisher, Braunschweig, Germany) or SERCA2 (IgG2a, mouse, 2A7-A1, Thermo Fisher) 1:200 in blocking solution (BS: 5% NGS, 5% BSA, 0.25% Triton-X in PBS) for 4 h at RT or overnight at 4 °C. The slices were washed three times with PBS for 5 min and incubated with the respective secondary antibody (goat anti-mouse IgG1 AF-488 (Thermo Fisher A-21121), GAM IgG1 AF-555 (Thermo Fisher A-21127), GAM IgG2a AF-555 (Thermo Fisher A-21137), goat anti-mouse IgG AF-647 (4410, Cell Signaling, Danvers, MA, USA) 1:400 in BS for 3 h at RT. After washing, the slices were incubated with wheat germ agglutinin (WGA-AF-647 Thermo Fisher W32466) 40 µg/mL and DAPI (3665, Roth, Karlsruhe, Germany) 1.67 μg/mL in PBS for 3 h. The slices were mounted with Fluoromount G (00-4958-02, Thermo Fisher; F4680 Sigma-Aldrich, Darmstadt, Germany) on a glass microscope slide, covered with a coverslip and dried for 1–7 days at 40–45% humidity.

### 4.4. Confocal Imaging

Two-dimensional confocal tile scans of 1 × 1–12 × 12 tiles with a pixel size of 0.1 × 0.1 µm^2^ and 10% tile overlap were obtained with a Zeiss LSM780 or Leica SP8, using a 63× oil immersion lens. Stitching of the tiles was performed with the respective microscope software package (ZEN or LAS X, respectively). All slices were imaged at the same depth of approximately 25–30 µm below the coverslip. A two-track imaging protocol was used for slices with 4–5 channels, with parallel excitation of DAPI and AF-555 with laser wavelengths of 405 nm and 561 nm, respectively, and a second track with laser wavelengths of 488 nm and 633 nm to excite FITC or AF-488 and AF-647, respectively. Slices with three channels (DAPI + RyR-AF488 + WGA-AF647 staining) were imaged in one sequence. For human tissue, we used an autofluorescence channel utilizing the 488 and 633 nm laser with a detection spectrum between 750 and 800 nm to detect lipofuscin and related autofluorescent granules [61].

### 4.5. Image Processing

Image tile scans were preprocessed by noise filtering and deconvolution with measured point spread functions. Subsequent image processing and analysis was performed with custom software based on published methods [2,31,32,62,63]. In brief, all microscopy channels, containing the signals stemming from DAPI, WGA, RyR and SERCA immunofluorescence, and dextran as well as lipofuscin were segmented by applying histogram-based local thresholds. Local thresholds were obtained by first using a box mean filter with an adjustable box size. If the signal intensity in the tile scan showed little region-dependent variation, a large box size was chosen (up to the size of the whole image). If the variation was greater, box sizes down to 100 × 100 µm^2^ were chosen. The result of the mean filter was subtracted from the original image. Within the resulting image, the local standard deviation *σ* and image mode *m* were calculated and the threshold *t* determined as *t* = *c σ* + *m*, with the factor *c* equaling 2 for DAPI, 3 for RyR, 1 for WGA, 0.5 for dextran 2 for SERCA and 4 for lipofuscin. Pixels with intensities ≥ *t* were considered the signal; otherwise, they were considered the background. From the resulting binary images of the signal, the binary lipofuscin signal was subtracted. Binary images were then median filtered with radius 1 and subjected to further analysis. Cell segments were created based on a watershed-transform run on the negated WGA distance map, as described [62]. The binary RyR image was morphologically dilated with radius 4, and the binary dextran image with radius 3. For each cell segment, the number of RyR-positive and dextran-positive pixels in the dilated images was counted and divided by the total number of pixels within the cell segment to obtain the respective fraction. If the RyR fraction was ≥0.15, the segment was classified as an RyR-positive cell. If the dextran fraction was ≥0.2, the segment was classified as a dextran-positive cell. Nuclei were detected and analyzed as described [32]. To extract and analyze the transverse tubular system (t-system), cell segments were morphologically closed and the difference between the closed and non-closed images identified as the t-system. The t-system density was calculated by counting the pixels of the t-system after skeletonization and dividing it by the total pixels of myocytes. The t-system distance was calculated as the mean value of the Euclidean t-system distance map within myocytes [31,37].

### 4.6. Statistics

Data are presented as mean ± standard error, if not otherwise indicated. Means between different groups were compared by the two-tailed Welch’s *t*-test or paired *t*-test as indicated. If more than one comparison was made within one figure, the resulting *p* values were corrected for multiple comparisons, using the Holm–Bonferroni method. Linear regression was performed with the built-in function fitlm of Matlab (version 2022b, MathWorks, Natick, MA, USA). The level of significance was set to α = 0.05. Cultured slices used for the dextran and RyR co-staining were not selected randomly but in such a way as to include slices with high and low contraction amplitudes. For the acquisition of images that were analyzed with respect to correlations with functional parameters, the researchers were blinded against groups and functional data.

## Figures and Tables

**Table 1:** Distribution parameters of the dextran and RyR signals in rabbit myocardial slices.

Fraction of	Median	Mean	95% Confidence Interval
Double-positive cells ^1^	3.4%	6.1%	3.5–8.6%
Double-positive pixels ^2^	1.2%	2.5%	1.3–3.7%
RyR in −dextran cells	9.4%	8.5%	6.7–10.3%
RyR in +dextran cells	1.4%	3.6%	2.1–5.1%
Dextran in −RyR cells	30.1%	34.0%	28.6–39.3%
Dextran in +RyR cells	4.1%	8.5%	5.2–12.0%

^1^ Fraction of cells is calculated in reference to the union of RyR- and dextran-positive cells; ^2^ fraction of pixels is calculated in reference to the union of RyR- and dextran-positive pixels.

**Table 2:** Distribution parameters of the dextran and RyR signals in human myocardial slices.

Fraction of	Median	Mean	95% Confidence Interval
Double-positive cells ^1^	0.39%	1.96%	0.09–3.84%
Double-positive pixels ^2^	0.53%	0.58%	0.36–0.80%
RyR signal in −dextran cells	1.4%	2.8%	6.7–10.3%
RyR signal in +dextran cells	0.15%	0.43%	0.16–0.71%
Dextran signal in −RyR cells	23.2%	24.7%	17.3–32.1%
Dextran signal in +RyR cells	4.5%	6.3%	3.0–9.6%

^1^ Fraction of cells is calculated in reference to the union of RyR- and dextran-positive cells; ^2^ fraction of pixels is calculated in reference to the union of RyR- and dextran-positive pixels.

**Table 3:** Test accuracy parameters of RyR immunofluorescence-based classification of dead and living cells when considering the dextran assay result as truth. PPV, positive predictive value; NPV, negative predictive value.

	Sensitivity	Specificity	NPV	PPV
rabbit, non-cultured	77.6%	18.9%	52.9%	58.2%
rabbit, cultured	69.1%	80.3%	75.2%	75.0%
human, non-cultured	69.5%	66.8%	30.6%	91.2%
human, cultured	45.0%	83.6%	48.1%	81.8%

## Data Availability

The data presented in this study are available on request from the corresponding author.

## Supplementary Materials

The supporting information can be downloaded at: https://www.mdpi.com/article/10.3390/ijms241713514/s1.
