# Supplementary material for: Ryanodine Receptor Staining Identifies Viable Cardiomyocytes in Human and Rabbit Cardiac Tissue Slices
Source: Int J Mol Sci. 2023 Aug 31;24(17):13514. doi: 10.3390/ijms241713514 (PMC10488113; doi:10.3390/ijms241713514)
Supplement: Supplementary file 1 [file ijms-24-13514-s001.zip › ijms-2572329-supplementary.pdf]

Table S1: Details of analyzed confocal tile scans

image-number	type	species	ID	Size of Tile Scan	Area [µm^2]	Living Myocytes in reference to whole image	Living Myocytes in reference to all cells	Dead Myocytes in reference to whole image	Dead Myocytes in reference to all cells	Overlap double-positive Myocytes to all D+/R+ Myocytes	Overlap double-positive Pixels to all D+/R+ Pixels
1	cultured	rabbit	R1	1x1	22380	23.60%	43.37%	30.81%	56.63%	0.01%	0.40%
2	cultured	rabbit	R1	1x1	22380	60.01%	78.77%	16.17%	21.23%	0.00%	0.14%
3	cultured	rabbit	R1	1x1	22380	36.72%	54.47%	30.79%	45.66%	0.13%	0.09%
4	cultured	rabbit	R1	7x7	888262	38.12%	50.31%	41.06%	54.19%	4.50%	0.79%
5	cultured	rabbit	R2	1x1	22380	53.99%	69.23%	30.52%	39.14%	8.36%	4.87%
6	cultured	rabbit	R2	1x1	22380	70.42%	83.07%	33.85%	39.93%	23.00%	4.97%
7	cultured	rabbit	R2	1x1	22380	43.70%	61.95%	34.01%	48.22%	10.17%	2.13%
8	cultured	rabbit	R2	1x1	22380	1.42%	2.60%	53.35%	97.40%	0.00%	0.52%
9	cultured	rabbit	R2	10x10	1812780	62.18%	78.20%	20.05%	25.21%	3.42%	1.76%
10	cultured	rabbit	R2	1x1	22380	5.86%	10.62%	49.36%	89.38%	0.00%	0.37%
11	cultured	rabbit	R2	3x3	163150	51.36%	77.30%	33.51%	50.44%	27.73%	9.31%
12	cultured	rabbit	R2	4x4	290045	57.58%	99.84%	0.35%	0.60%	0.44%	1.19%
13	cultured	rabbit	R2	1x1	22380	67.48%	86.08%	16.79%	21.41%	7.50%	13.89%
14	cultured	rabbit	R2	10x10	1812780	71.87%	92.75%	7.59%	9.79%	2.54%	3.43%
15	cultured	rabbit	R2	1x1	22380	40.23%	71.48%	30.45%	54.10%	25.59%	15.42%
16	cultured	rabbit	R2	1x1	22380	62.53%	76.65%	28.73%	35.21%	11.86%	5.59%
17	cultured	rabbit	R3	10x10	1812780	45.12%	90.69%	7.64%	15.36%	6.05%	3.48%
18	cultured	rabbit	R3	10x10	1812780	0.57%	1.14%	48.86%	98.95%	0.10%	0.25%
19	cultured	rabbit	R3	5x5	453195	28.45%	37.33%	49.35%	64.75%	2.08%	1.76%
20	cultured	rabbit	R4	9x9	852007	0.69%	1.25%	54.61%	99.52%	0.77%	0.31%
21	cultured	rabbit	R4	8x8	1160179	62.47%	84.84%	11.90%	16.16%	1.00%	0.52%
22	cultured-Saponin-EGTA	rabbit	R5	8x8	1160179	22.13%	42.58%	47.67%	91.71%	34.29%	9.25%
23	cultured	rabbit	R5	6x6	652601	27.15%	39.82%	45.57%	66.83%	6.65%	0.75%
24	cultured	rabbit	R5	3x3	163150	42.45%	65.02%	24.94%	38.20%	3.22%	0.45%
25	cultured	rabbit	R6	12x12	2610403	34.45%	69.29%	21.44%	43.13%	12.42%	4.34%
26	cultured	rabbit	R6	12x12	2610403	22.93%	33.21%	48.11%	69.69%	2.90%	1.18%
27	cultured	rabbit	R6	12x12	2610403	0.03%	0.04%	68.68%	99.96%	0.00%	0.05%
28	cultured	rabbit	R6	12x12	2610403	0.01%	0.01%	75.26%	99.99%	0.00%	0.09%
29	cultured	rabbit	R6	12x12	2610403	3.14%	4.29%	72.51%	99.29%	3.58%	1.36%
30	cultured	rabbit	R6	12x12	2610403	57.65%	85.01%	12.55%	18.51%	3.52%	1.22%
31	cultured	rabbit	R7	12x12	2610403	7.54%	11.00%	68.10%	99.41%	10.41%	1.46%
32	cultured	rabbit	R7	12x12	2610403	66.09%	94.59%	4.44%	6.36%	0.94%	0.17%
33	cultured	rabbit	R7	12x12	2610403	4.81%	7.04%	67.59%	98.98%	6.03%	1.06%
34	cultured	rabbit	R7	12x12	2610403	17.16%	22.23%	64.12%	83.05%	5.28%	1.14%
35	cultured	rabbit	R7	12x12	2610403	17.58%	25.68%	51.39%	75.08%	0.76%	0.16%
36	cultured	rabbit	R7	12x12	2610403	62.61%	80.29%	31.71%	40.67%	20.96%	2.20%
37	fresh	rabbit	R8	12x12	2610403	53.37%	75.23%	38.22%	53.87%	29.09%	4.82%
38	fresh	rabbit	R8	8x8	1160179	65.04%	98.16%	22.36%	33.75%	31.91%	5.77%
39	fresh	rabbit	R8	7x7	888262	53.30%	96.78%	38.18%	69.33%	66.12%	6.44%
40	fresh	rabbit	R8	7x7	888262	36.45%	80.03%	22.65%	49.74%	29.77%	4.55%
41	cultured	human	H1	6x6	652601	9.44%	48.70%	9.96%	51.43%	0.13%	0.23%
42	cultured	human	H1	6x6	652601	0.94%	2.69%	34.07%	97.31%	0.00%	1.56%
43	cultured	human	H1	6x6	652601	0.91%	2.48%	35.59%	97.56%	0.05%	0.52%
44	cultured	human	H1	6x6	652601	22.55%	64.33%	13.56%	38.70%	3.04%	0.79%
45	cultured	human	H1	6x6	652601	4.11%	9.06%	41.42%	91.32%	0.38%	0.24%
46	cultured	human	H1	6x6	652601	15.37%	29.55%	36.85%	70.84%	0.39%	0.21%
47	cultured	human	H1	6x6	652601	34.21%	55.30%	34.07%	55.07%	10.37%	0.64%
48	fresh	human	H2	4x4	290045	27.50%	98.42%	22.87%	81.87%	80.29%	15.30%
49	fresh	human	H2	4x4	290045	21.95%	67.09%	29.89%	91.34%	58.43%	10.92%
50	fresh	human	H2	6x6	652601	30.10%	71.92%	13.78%	32.92%	4.84%	2.37%
51	fresh	human	H2	6x6	652601	25.60%	77.90%	15.40%	46.86%	24.75%	4.33%
52	fresh	human	H3	6x6	652601	73.12%	96.64%	21.67%	28.64%	25.28%	3.06%
53	fresh	human	H3	6x6	652601	61.36%	94.81%	30.24%	46.72%	41.53%	7.08%
54	fresh	human	H3	6x6	652601	73.12%	98.74%	5.11%	6.91%	5.65%	1.37%
55	fresh	human	H4	6x6	652601	23.81%	58.90%	21.00%	51.95%	10.85%	2.17%
56	cultured	human	H3	6x6	652601	0.38%	1.04%	36.05%	99.16%	0.20%	0.49%
57	cultured	human	H3	6x6	652601	11.26%	15.69%	63.94%	89.08%	4.77%	0.58%
58	cultured	human	H3	3x3	163150	3.28%	5.75%	54.69%	95.99%	1.75%	0.55%
59	cultured	human	H3	5x5	453195	2.12%	3.86%	53.02%	96.69%	0.55%	0.54%
60	fresh	human	H4	6x6	652601	51.59%	97.03%	10.82%	20.35%	17.38%	5.37%
61	fresh	human	H4	6x6	652601	32.44%	79.01%	27.18%	66.18%	45.20%	8.71%
62	fresh	human	H3	6x6	652601	72.35%	97.75%	11.71%	15.82%	13.56%	3.34%
63	fresh	human	H3	6x6	652601	61.02%	85.18%	35.84%	50.04%	35.23%	4.72%

Table S1. Fractions of analyzed tile scans

Table S2: Test accuracy parameters

**rabbit, non-cultured**	classification based on RyR	
	RyR-positive	RyR-negative
dextran-negative (living)	0.43	0.12
dextran-positive (dead)	0.31	0.14
ground truth		

**rabbit, cultured**	classification based on RyR	
	RyR-positive	RyR-negative
dextran-negative (living)	0.32	0.14
dextran-positive (dead)	0.11	0.43
ground truth		

**human, non-cultured**	classification based on RyR	
	RyR-positive	RyR-negative
dextran-negative (living)	0.58	0.25
dextran-positive (dead)	0.06	0.11
ground truth		

**human, cultured**	classification based on RyR	
	RyR-positive	RyR-negative
dextran-negative (living)	0.28	0.34
dextran-positive (dead)	0.06	0.32
ground truth		

Table S2. Confusion matrices of the four different slice groups investigated in this study. The dextran-signal was considered as ground truth, with dextran-positive cell segments belonging to dead cells and dextran-negative cell segments beloning to living cells. The RyR signal was used as a classifier to predict living cells. The colors are chosen to reflect the appearance in the confocal images: *red*, true positive (living) myocytes; *green*, true negative (dead myocytes); *yellow*, false positive (classified as living but actually dead or dying myocytes), gray or uncolored, false neative (classified as dead but actually living myocytes).

Figure S1: Tile scans solely stained for RyR

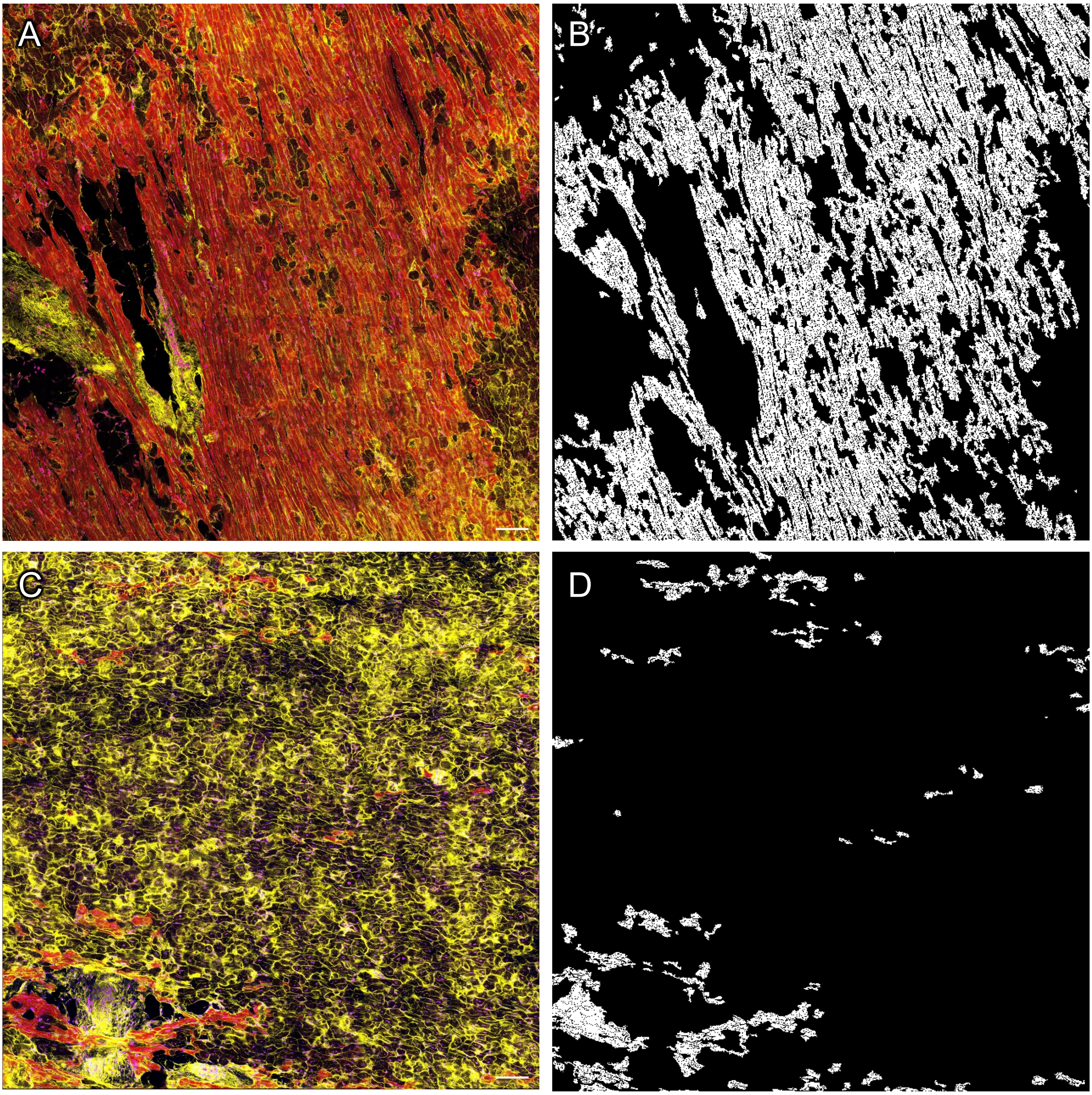

Figure S1. Two-dimensional confocal tile scans of rabbit slices stained for RyR (*red*), with WGA (*yellow*) and DAPI (*blue*). The tissue was cultured for 6 days. (**A**) Example of a slice with abundant RyR staining. (**B**) Corresponding segmentation of (A): RyR positive cells displayed in *white*. (**C**) Example of a slice with nearly absent RyR staining. (**D**) Corresponding segmentation of (C). Scale bar length is 100 µm.

Figure S2: Test characteristics

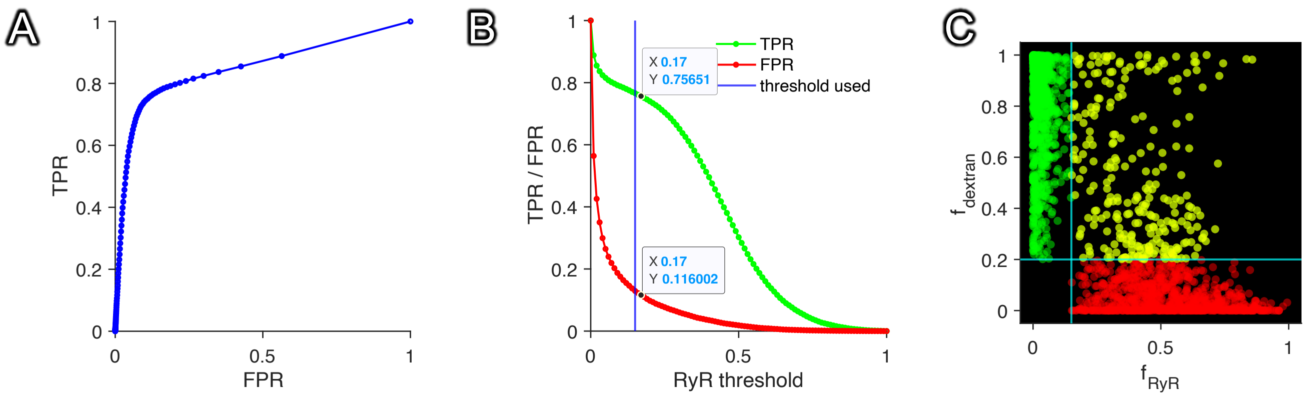

Figure S2. Test and thresolding characteristics in data collected from four confocal tile scans of cultured rabbit slices. The fractions of RyR- and dextran-positive pixels from each cell segment contained in the 34 images were used to classify the segments as RyR-positive or -negative as well as dextran-positive or -negative. (**A**) Receiver-Operating-Characteristics (ROC), showing the true positive rate (TPR, = sensitivity) over the false positive rate (FPR, = 1-specificity). To obtain the data, the RyR fraction threshold for classifying a cell segment as RyR-positive was varied from 0 to 1 in steps of 0.01. As ground truth, the dextran signal was used with a threshold of 0.2, above which cell segments were considered as dead, otherwise as viable cells. (**B**) TPR (*green*) and FPR (*red*) over the RyR threshold. The blue line indicates the threshold used in this study. (**C**) Scatter plot showing each cell segment as a circle and its fraction of RyR-positive (f_{RyR}) and dextran-positive ($f_{dextran}$) pixels. For clarity, the data of only four randomly selected images are shown. The two lines (cyan) indicate the f_{RyR} and $f_{dextran}$ thresholds used throughout the study. With the dextran-based classification as ground truth, colors indicate true positive living cells (*red*), true negative dead cells (*green*) and false positive dead cells (*yellow*). False negative cells are not shown. Total number of cell segments in the four images: 2161, RyR-positive: 1115, dextran-positive: 1205, double-positive: 273.

Figure S3: Tile scan co-stained with dextran and for SERCA

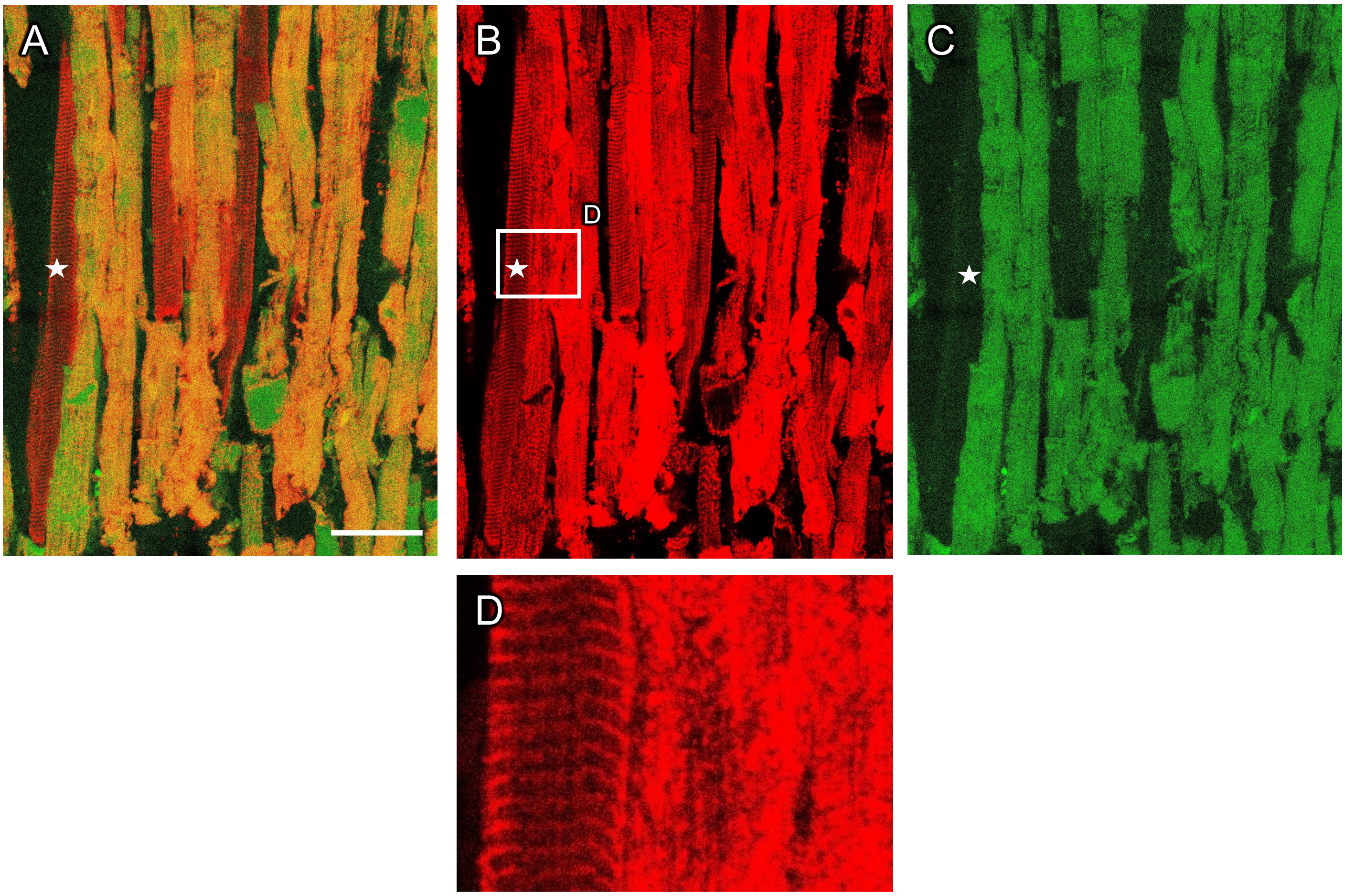

Figure S3. Two-dimensional tile scans of rabbit co-stained for SERCA (*red*) and dextran (*green*).
(**A**) Overlay of SERCA and dextran signals showing dextran-positive cells that also contain SERCA signal (appearing *yellow*). A dextran-negative cell with regularly aligned structure of the SERCA signal (z-line pattern) is highlighted (*star*), while in dextran-positive cells the SERCA pattern is irregular. (**B**) SERCA signal only. (**C**) Dextran signal only. (**D**) Magnification of the region indicated in (B). Scale bar length in (A) is 50 μm and also applies to (B, C).
